# Editorial: Furthering precision medicine and cancer prevention through novel insights in molecular and chemical carcinogenesis

**DOI:** 10.3389/fonc.2024.1496908

**Published:** 2024-10-08

**Authors:** William H. Bisson, Karen T. Liby, Jamie J. Bernard

**Affiliations:** ^1^ Integrative Toxicology and Cancer Prevention, Durham, NC, United States; ^2^ Indiana University School of Medicine, Department of Medicine Division of Hematology/Oncology, Indianapolis, IN, United States; ^3^ Department of Pharmacology and Toxicology, Michigan State University, East Lansing, MI, United States; ^4^ Department of Medicine, Division of Dermatology, Michigan State University, East Lansing, MI, United States

**Keywords:** precision, prevention, carcinogenesis, chemical, mechanisms, technology

A healthy lifestyle, pharmacological strategies, or decreased exposure to environmental carcinogens can reduce risk or delay the development of cancer (1). Several decades of research have explored mechanisms of carcinogenesis, characterized carcinogenicity hazards, and identified novel targets for intervention. Many of these integral discoveries contributed to the identification of the 15 hallmarks of cancer and the 10 key characteristics of carcinogens, KCCs (2–4) Leveraging these findings has the potential to foster the design and the implementation of precision prevention strategies on multiple levels (5).

This Research Topic aimed to produce a collection of articles that discuss known and novel biological targets and biomarkers *in vitro*, *in vivo*, and in human cohorts, including the emerging role of the cancer hallmarks phenotypic plasticity and circulating cell-derived biomarkers. The importance of integrating mechanistic and epidemiological data-driven approaches was highlighted. Particularly, these articles focused on identified early mechanisms of molecular and chemical carcinogenesis aiming to inform precision prevention and to reduce the burden of cancer health disparities.

The complexity of cancer requires a comprehensive approach to understand its diverse manifestations and underlying mechanisms. The Research Topic begins with the perspective article Senga et al. highlighting the need to integrate clear endpoints that anchor KCCs to the acquisition of a complete malignant phenotype into chemical testing. Thus, an all-encompassing strategy that incorporates both evolving KCCs and cancer hallmarks, including the role of the microenvironment, is essential to enable the targeted identification of prevalent carcinogens and facilitate zone-specific prevention strategies. To achieve this goal, collaboration between the KCCs and cancer hallmark communities becomes essential.

Schroeder et al. demonstrated that environmental chemicals with established exposure disparities between non-Hispanic Black women and non-Hispanic White women may influence breast phenotypic plasticity, a new hallmark of cancer. This type of plasticity is associated with basal-like breast cancers typically associated with an aggressive triple-negative subtype which affects African American women at rates of 2-3 times that of White women. These data were largely generated with a high-content imaging microscope demonstrating that innovative techniques will bring us closer to understanding cancer disparities at a molecular level. In addition, to better address and understand breast cancer disparities, it is also important to understand risk among different races. Most epidemiological studies have been performed on non-Hispanic White women. Original research by Patil et al. studied benign breast disease and the subsequent development of breast cancer in African American women.

The identification of extracellular vesicles (EVs) *in vivo* holds potential for the discovery of early biomarkers of carcinogenesis, and/or toxicity endpoints. EVs from donor cells communicate with recipient cells and/or tissues and have the potential to induce toxicity or promote tumorigenesis. However, much of what is currently known about EVs is from studies that perform exogenous administration. A perspective by Nambiar et al. reviews methodologies that track and alter EVs directly *in vivo*, as they are released by donor cells. The authors make the argument that advancements in EV engineering with mouse transgenesis and modern sequencing technologies may provide more insight into this largely unknown area of native EV function and biology. A perspective article by Silver et al. highlights the significance of detecting and characterizing circulating EVs as biomarkers for chemotherapy-induced cardiotoxicity. There are current capabilities to characterize circulating EVs in mice and human liquid cohorts for translational toxicology and cancer medicine.

The perspective article by Hariharan et al. discusses the significance of using human data-driven approaches to improve wellness and reduce tumor recurrence in cancer survivors. Regaining wellness is challenging due to the presence of a myriad of issues induced by radiation, chemotherapy, immunotherapy and/or targeted interventions. This perspective provides promising evidence that global interventions may be possible with data-driven approaches coupled to quality of life and cognitive measurements as well as biological age measurements.

Two original articles examined molecular and carcinogenic effects of a chemical exposure.

Carswell et al. used targeted templated Oligo-sequencing and DNA methylation profiles on a genome-wide scale to identify DNA methylation alterations with early-in-life dichloroacetic acid (DCA) exposure to determine potential mechanisms of liver tumorigenesis. This question arose from previous paradoxical findings whereby DCA exposure shunted cellular metabolism from aerobic glycolysis, which is termed the Warburg Effect and is associated with cancer, to oxidative phosphorylation. Gonzalez-Pons and Bernard examined the interactions between a high-fat diet and benzo(a)pyrene on tumorigenesis in an aggressive mouse model of estrogen receptor negative breast cancer.

Cohen provides a comprehensive review of the different modes of action of chemical carcinogenesis and how to screen for human carcinogens using the primary endpoints of DNA reactivity and mutagenesis, immunomodulation, increased estrogenic activity, and cytotoxicity with consequent regeneration. Mechanistic understanding of chemical carcinogenicity and hazard identification and evaluation will be critical for prevention of future cancers.

In conclusion, cancer prevention strategies will significantly contribute to reaching the goal of the White House Cancer Moonshot Initiative of decreasing 50% cancer mortality by the year 2047 (6). This can be only achieved by bringing together novel ideas, multidisciplinary efforts, and cutting-edge technologies. Hence, this Research Topic provides a collection of articles that will encourage further research and collaborative efforts in the fields of molecular and chemical carcinogenesis for precision prevention and environmental health.
